# Gut microbiota-derived metabolites as novel therapies for inflammatory bowel diseases: Role of nuclear receptors

**DOI:** 10.1016/j.fmre.2024.01.018

**Published:** 2024-02-08

**Authors:** Feng Li, Xiaokang Wang, Yuting Cai, Yanke Lin, Ying Tang, Shuai Wang

**Affiliations:** a Guangzhou Eighth People's Hospital, Guangzhou Medical University, Guangzhou 510440, China; b Department of Pharmacy, Shenzhen Longhua District Central Hospital, Shenzhen 518110, China; c School of Pharmaceutical Sciences, Guangzhou University of Chinese Medicine, Guangzhou 510006, China; d Guangdong TCRCure Biopharma Technology Co., Ltd, Guangzhou 510030, China; e Science and Technology Innovation Center, Guangzhou University of Chinese Medicine, Guangzhou 510405, China

**Keywords:** Inflammatory bowel diseases, Gut microbiota-derived metabolites, Nuclear receptors

## Abstract

Inflammatory bowel diseases (IBDs) are increasingly recognized as a pressing global health concern. The gut microbiome emerges as both a potential therapeutic target and a repository for pharmacological interventions in IBDs management. This perspective aims to elucidate the pivotal findings from recent studies concerning the anti-inflammatory properties of gut microbiota-derived metabolites (GMDMs), dissect the strengths and challenges of GMDMs as treatment strategies for IBDs, and highlight the integral role of nuclear receptors in mediating the interplay between IBD pathogenesis and GMDMs. Through the integration of these perspectives, our objective is to deepen the understanding of the therapeutic promise of nuclear receptor-targeted GMDMs, thus propelling forward the exploration and formulation of new pharmacological treatments for IBDs.

## 1 Introduction

Inflammatory bowel diseases (IBDs), which include ulcerative colitis and Crohn's disease have become a global emerging disease [1]. IBDs mainly occur in developed countries previously, whereas their incidence has gradually increased in developing countries recently. The pathogenesis of IBDs is tightly associated with genetic susceptibility, environmental factors, immune dysfunction, and gut microbiota. Although the mechanisms for IBDs development are well-investigated, IBDs cannot be completely cured by available medical or surgical therapy [2]. For instance, anti-inflammatory agents are generally used in the first step toward IBDs therapy, but they are associated with a multitude of side effects. Therefore, novel molecules against IBDs require further exploration. Changes in the composition and diversity of the gut microbiota in IBDs patients are linked to the pathogenesis of IBDs [1]. Owing to a critical role in the pathogenesis of IBDs, gut microbiota-derived metabolites (GMDMs) act as a promising drug reservoir for IBDs.

## 2 Role of GMDMs in IBDs

Accumulating shreds of evidence have implicated an array of GMDMs, most of which are identified by using combining approaches including functional metagenomics, host reporter assays, computational chemistry, synthetic biology, and bioinformatics. GMDMs exert a variety of pharmacological activities *in vivo*, thereby regulating the development of diverse diseases (e.g., inflammatory diseases, metabolic disorders, cancers, Alzheimer's disease, and cardiovascular diseases).

Several types of GMDMs including bile acid derivatives, SCFA, and tryptophan metabolites have drawn intense research attention owing to their associations with IBDs. Firstly, the general changes in bile acid composition (increased abundance of primary bile acids, with a corresponding reduction in secondary bile acids) have occurred in IBDs patients [3]. In turn, the abundance of bile acids plays a significant role in IBDs development. Lower fecal deoxycholic acid abundance was associated with more severe intestinal inflammation in IBDs patients [4]. Besides, anti-inflammatory SCFAs, reduced in IBDs patients, regulate the immune function and avoid an excessive immune response, thereby having a positive clinical impact on IBDs patients [5]. Moreover, lower serum tryptophan levels and higher tryptophan metabolites (i.e., quinolinic acid) were found in IBDs patients than in healthy controls, which was attributed to activation of the kynurenine pathway. Accumulating pieces of evidence indicate a negative correlation between serum tryptophan levels (lower levels in IBDs patients than in controls) and IBDs severity. Tryptophan deficiency promotes the development of IBDs, evidenced by aggravating disease activity [6,7]. Furthermore, medium-chain fatty acids or succinate also play important roles in IBDs development [8]. Replacing n-6 fatty acids with medium-chain triglycerides caused a decreased incidence of spontaneous colitis in mice [9]. These findings indicate that GMDMs could act as a new therapeutic approach for restoring intestinal function and a promising strategy for IBDs treatment.

## 3 Nuclear receptors link GMDMs and IBDs

**Fig. 1:**
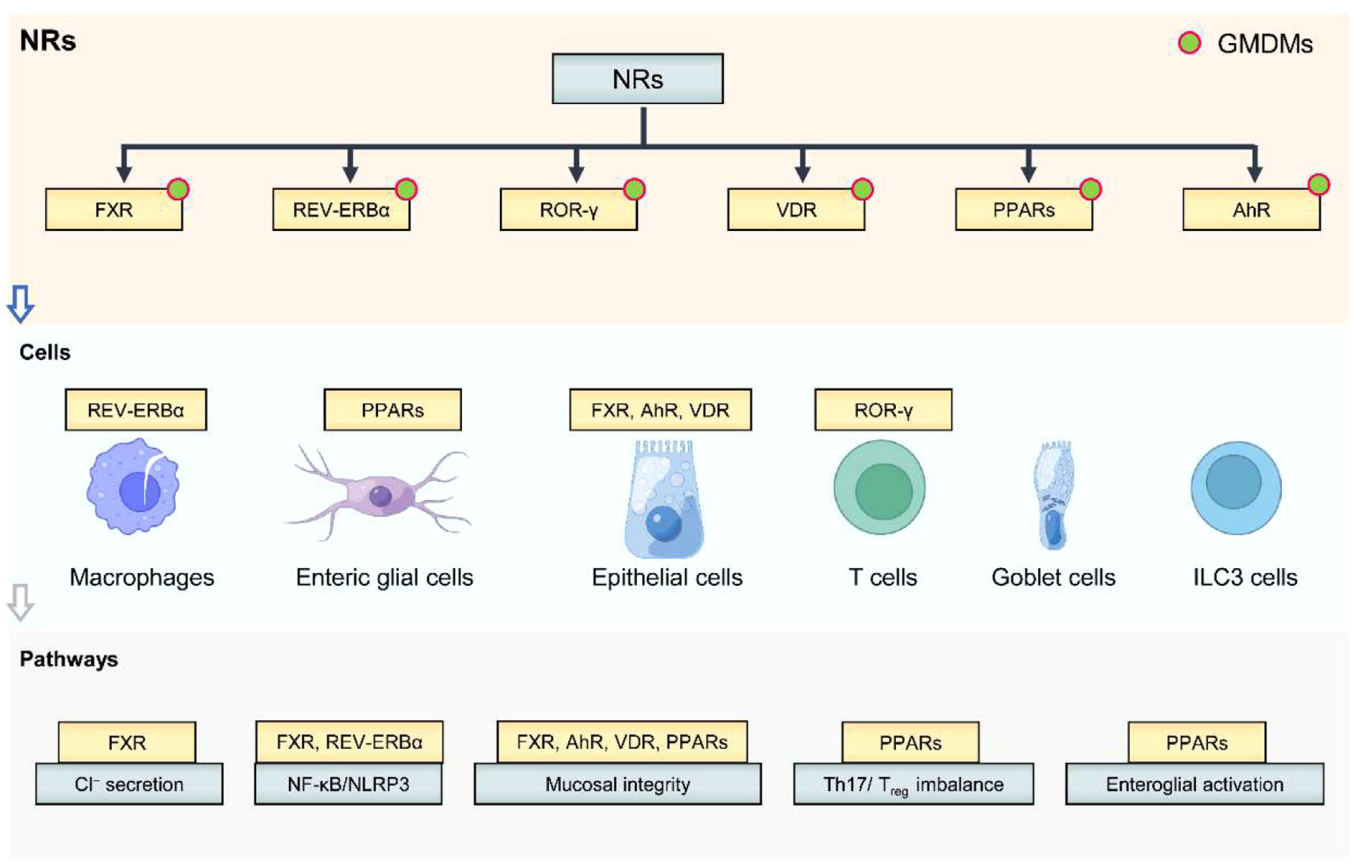
**The role of nuclear receptors in the regulation of IBDs.** Nuclear receptors regulate colitis through diverse regulatory mechanisms in specific cells (e,g., macrophages, enteric glial cells, epithelial cells, T cells, goblet cells, and ILC3 cells. The regulatory mechanisms include Cl^−^ secretion, NF-κB/NLRP3 pathway, Mucosal integrity, Th17/T_reg_ imbalance, and enteroglial activation. These nuclear receptors can be targeted by GMDMs. ILC3, Innate lymphoid cells type 3.

Most recent years of studies uncover a significant role of nuclear receptors (i.e., FXR [10], REV-ERBα [11,12], RORγ [13,14], VDR [15], AhR [16], and PPAR-α [17,18] in IBDs. Alterations in signaling pathways, cytokine production, immune cell responses, autophagy, gut microbiome, and intestinal barrier function are involved in the mechanisms by which nuclear receptors regulate gut inflammation (Fig. 1). The biological mechanisms occur in various immune-related cells in the body such as macrophages, enteric glial cells, epithelial cells, T cells, goblet cells, and ILC3 cells (Fig. 1). The FXR is a nuclear receptor that is primarily activated by bile acids and plays a crucial role in the direct regulation of gene expression related to bile acid synthesis, lipid metabolism, and glucose homeostasis. FXR regulates gut inflammation through distinct and intertwined mechanisms including cytokine production/release in innate immune cells, gut barrier function, and intestinal stem cell proliferation. A previous study demonstrates that FXR activation attenuates Ca^2+^ and cAMP-dependent Cl^−^ secretory responses in colonic epithelial cells [10]. NF-κB/NLRP3 appears to be a critical signaling pathway involved in the regulation of colitis. Expression of p65 (an NF-κB subunit) and NLRP3 inflammasome is decreased upon FXR activation. REV-ERBα is a nuclear receptor that is activated by endogenous heme, and functions as a transcriptional repressor with a pivotal role in the circadian regulation of inflammation and metabolism. We found the activation of the nuclear receptor REV-ERBα alleviates experimental colitis by inhibiting the NF-κB/NLRP3 pathway [11]. In addition to the aforementioned pathways, a diverse array of nuclear receptors intricately regulates the pathophysiology of IBDs through a spectrum of regulatory mechanisms at epigenetic, transcriptional, and post-transcriptional levels (Fig. 1).

**Fig. 2:**
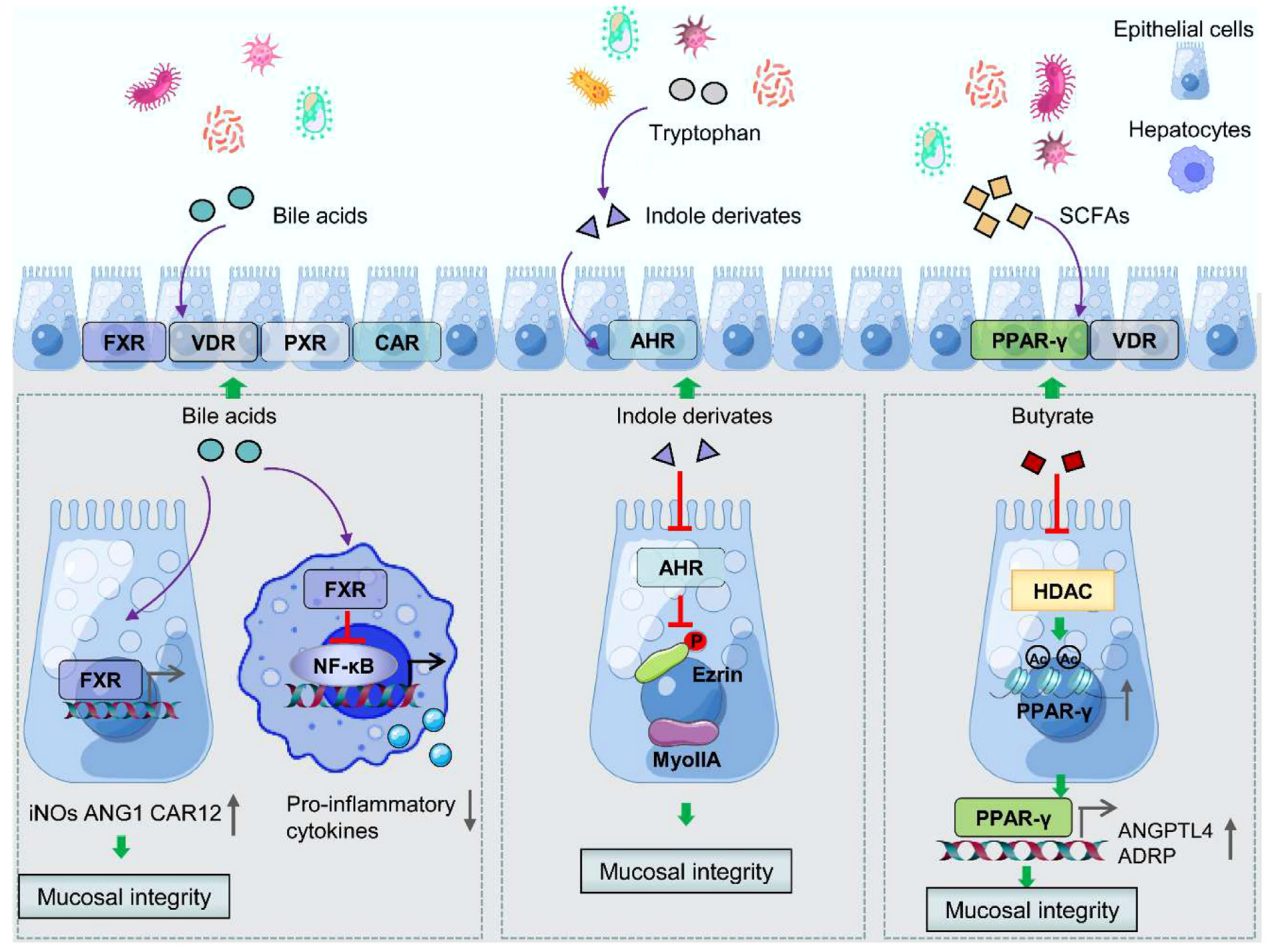
**The regulatory mechanisms of anti-IBD effects of GMDMs through nuclear receptors targeting**. GMDMs such as bile acids, indole derivatives, and SCFAs significantly maintain the intestinal barrier integrity or suppress release of pro-inflammatory cytokines via interacting with nuclear receptors in gut epithelial and immune cells, thus protecting the host from colitis.

Interactions between gut microbiota and nuclear receptors are important mechanisms for GMDM-controlled IBDs development. To date, FXR, REV-ERBα, RORγ, AhR, VDR, and PPARs have been regarded as critical bridges between GMDMs and IBDs (Fig. 1). Bile acids bind to nuclear receptors and regulate immune responses in IBDs [23]. For example, bile acids activate FXR and increase the levels of iNOs, ANG1, and CAR12, which are involved in anti-bacterial defense and maintaining the integrity of the intestinal epithelial barrier, thereby impacting IBDs development (Fig. 2). Activation of FXR by bile acids also inhibits the release of pro-inflammatory factors (i.e., IL-6, IL-1, and TNF-α) in macrophages [19]. Indole derivatives such as indoleacetic acid, indole-3-acetaldehyde, and indole-3-aldehyde are tryptophan metabolites obtained from the gut microbiota. Some of the indole derivatives act as agonists for AhR and are involved in IBDs pathogenesis by protecting the intestinal barrier through the activation of Ezrin and Myosin IIA [20] (Fig. 2). PPARγ is a nuclear receptor that is activated by fatty acids and eicosanoids, and it directly regulates gene expression, playing a key role in the inflammatory response. The SCFA butyrate modulates the epithelial damage and regulates IBDs through activation of PPARγ and up-regulation of angiopoietin-like protein 4 (ANGPTL4)/adipose differentiation-related protein (ADRP) expressions [21] (Fig. 2).

GMDMs such as SCFAs, bile acids, and amino acid derivatives, are products of microbial fermentation and metabolism that act as signaling molecules. The commonality among gut microbiota metabolites in the context of treating diseases is their anti-inflammatory action mediated through nuclear receptors. These metabolites, regardless of their diverse structures and origins, converge on a shared pathway of modulating nuclear receptors, which are pivotal in controlling gene expression related to inflammation and immune responses. The specificity of gut microbiota metabolites in treating diseases is attributed to the unique interactions they have with different nuclear receptors, which can vary based on the disease context. For instance, the activation of the AhR by tryptophan metabolites is particularly relevant in the context of autoimmune diseases due to its role in maintaining intestinal immune tolerance.

Immune maturation, immune homeostasis, host energy metabolism, and maintenance of mucosal integrity are tightly associated with the development of IBDs [22]. Regulation of immune cells (i.e., T cells, B cells, and macrophages) by GMDM-nuclear receptor axis is a key downstream mechanism for the anti-IBD effects. For instance, SCFAs modulate the maintenance of mucosal integrity by expanding T_reg_ cell proportions, and SCFAs reduction results in intestinal injury in IBDs patients. Similarly, the reduction of gut microbiota diversity reduced the SCFAs production and induced an imbalance between Th17 and T_reg_ cells, thus promoting intestinal inflammation in IBDs [23]. Besides, GMDMs control the transcription of inflammatory factors through epigenetic regulation. For example, butyrate, a known SCFA, affects pro-inflammatory genes (i.e., *Il-6* and *Nos2*) and intestinal immune homeostasis via inhibiting histone deacetylase [24].

## 4 Therapeutic potential of nuclear receptor targeting GMDMs for IBDs

The therapeutic landscape of IBDs is witnessing a great change with the emergence of gut microbiota metabolites as modulators of nuclear receptors. These metabolites, products of the intricate symbiosis between the host and its resident microbiota, are being recognized for their ability to influence host immunity and inflammatory pathways. Clinically, several gut microbiota metabolites have either been adopted or are under investigation in clinical trials. Tryptophan metabolites, including indole and its derivatives, have demonstrated promise in clinical trials [25]. In a randomized clinical trial, butyrates are effective in the treatment of pediatric obesity [26]. The advantages of using gut microbiota metabolites in IBDs treatment include their high biocompatibility, low toxicity, and the ability to target the gut directly, potentially reducing systemic side effects. Another strength of GMDMs is that they serve as a key factor in host-microbiota cross-talk. These molecules regulate energy metabolism and immune response by linking the gut and other tissues (e.g., liver and brain) through blood circulation. In a circulating manner, they modulate energy metabolism and immune homeostasis by interacting with biological targets in tissues involved in the physiological functions and pathogenesis of IBDs. Challenges associated with the drug development of GMDMs may include functional stability, safety problems (side effects), clinical potency, and pharmacokinetics properties. Moreover, challenges such as inter-individual efficacy variability and stability within the complex gut environment may limit their therapeutic utility.

## 5 Conclusion

In this perspective, we present a review on the robust interconnection between GMDMs and IBDs. We explore the bidirectional relationship wherein IBDs influence the concentrations of GMDMs, while these metabolites, in turn, act as significant modulators in the pathogenesis of IBDs.We highlight several specific GMDM classes such as bile acids, SCFAs, and tryptophan metabolites that participate in the progression of IBDs by interacting with nuclear receptors. This knowledge allows for a better understanding of the strength of nuclear receptor-targeted GMDMs, which can be identified as a promising therapeutic approach for IBDs in the future.

## Declaration of competing interest

The authors declare that they have no conflicts of interest in this work.
